# Supplementary material for: Extension of human lncRNA transcripts by RACE coupled with long-read high-throughput sequencing (RACE-Seq)
Source: Nat Commun. 2016 Aug 17;7:12339. doi: 10.1038/ncomms12339 (PMC4992054; doi:10.1038/ncomms12339)
Supplement: Supplementary Information — Supplementary Figures 1-10 and Supplementary Tables 1-5 [file ncomms12339-s1.pdf]

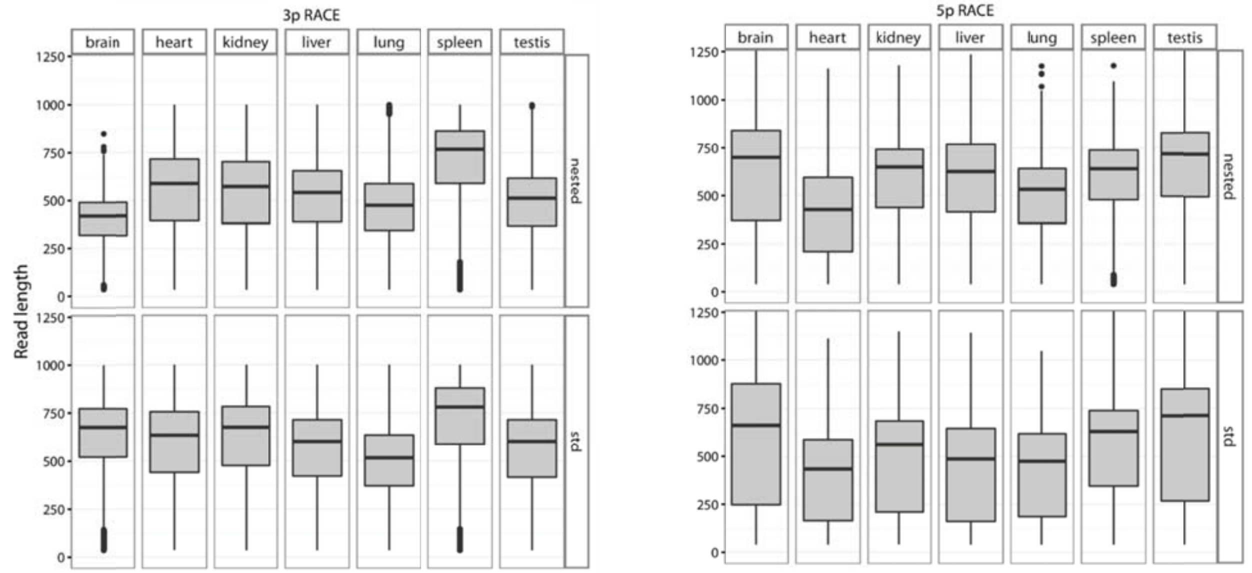

Supplementary Figure 1: Box-plots of read length distributions across seven tissues targeted by 5' and 3' standard ("std") and nested RACE-Seq.

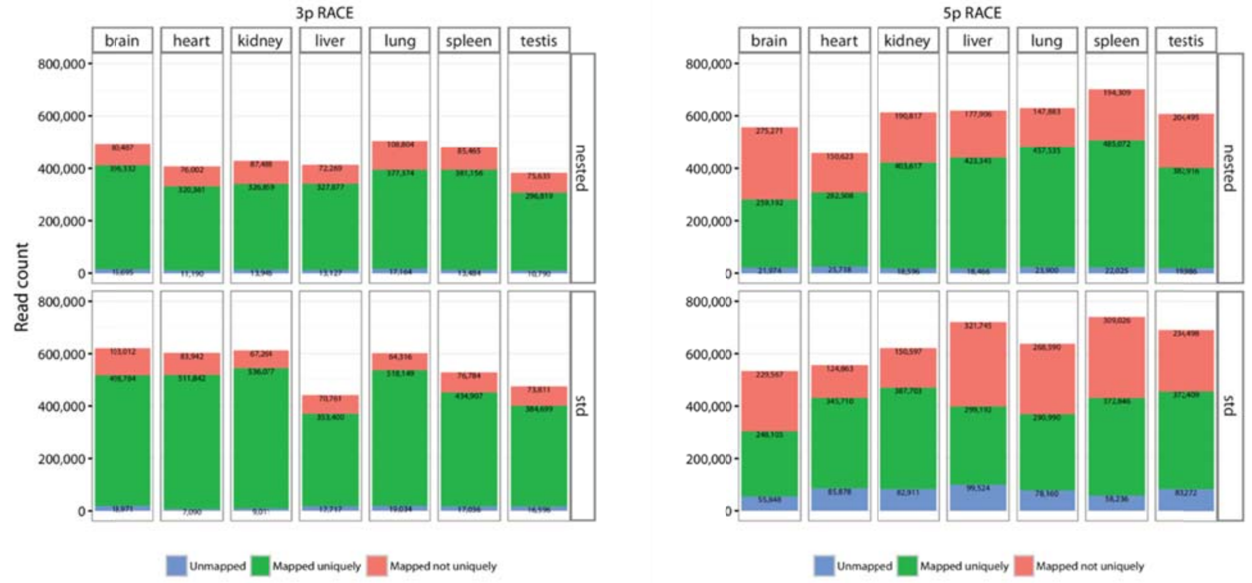

Supplementary Figure 2: Genome mapping statistics. Bar plot showing the number of 454 reads that were unmapped (blue) mapped uniquely (green) and multiple times (coral) to the reference human genome (GRCh37).

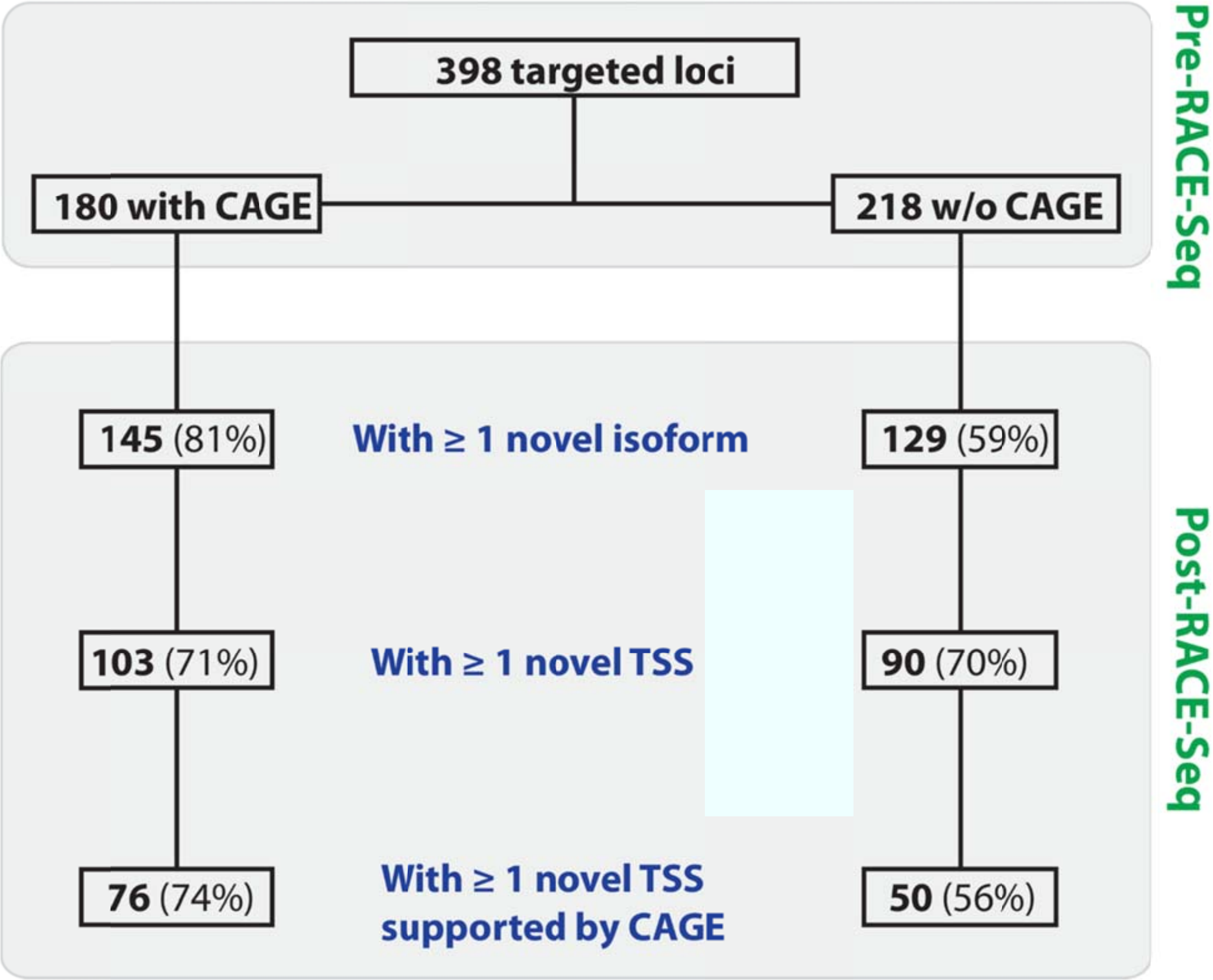

Supplementary Figure 3: Flowchart explaining the CAGE enrichment analysis and summarized results.

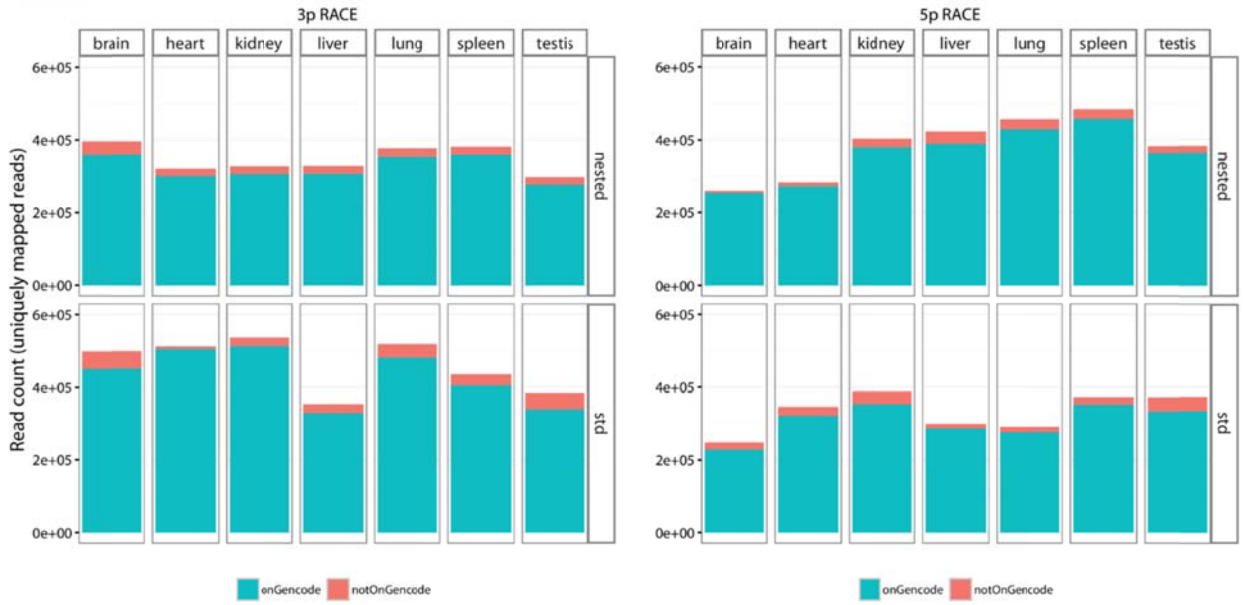

Supplementary Figure 4: Read mapping statistics. Number of uniquely mapped 454 reads overlapping GENCODE-annotated loci (green), vs. in intergenic regions (coral).

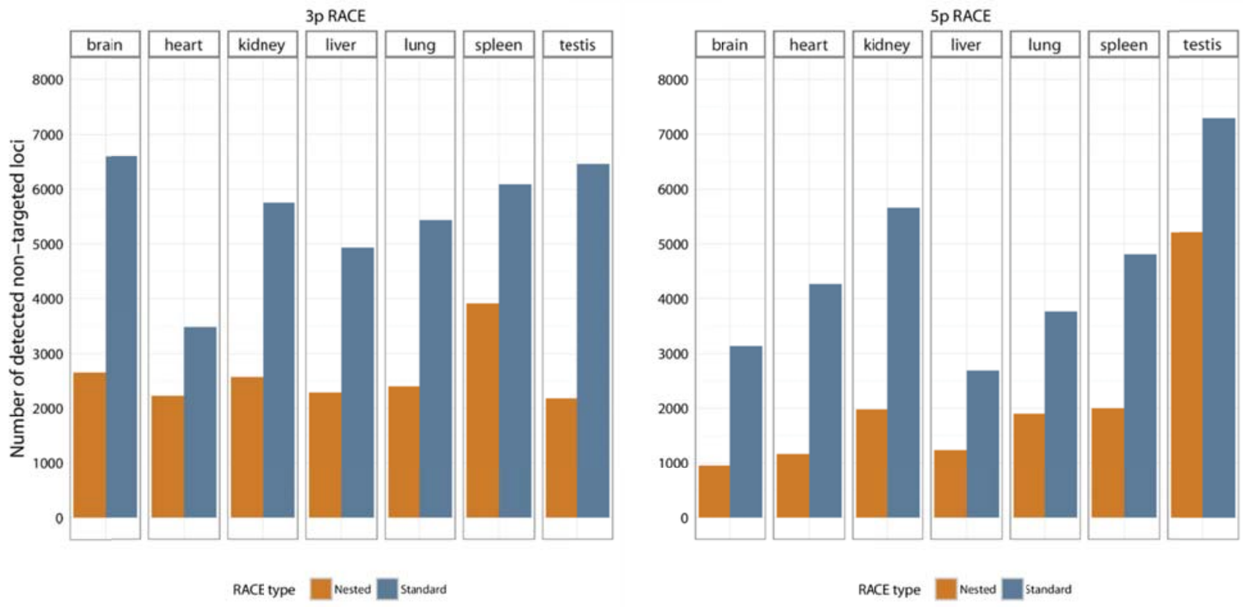

Supplementary Figure 5: Number of amplified non-targeted loci in nested and standard RACE-Seq in the seven tissues assayed.

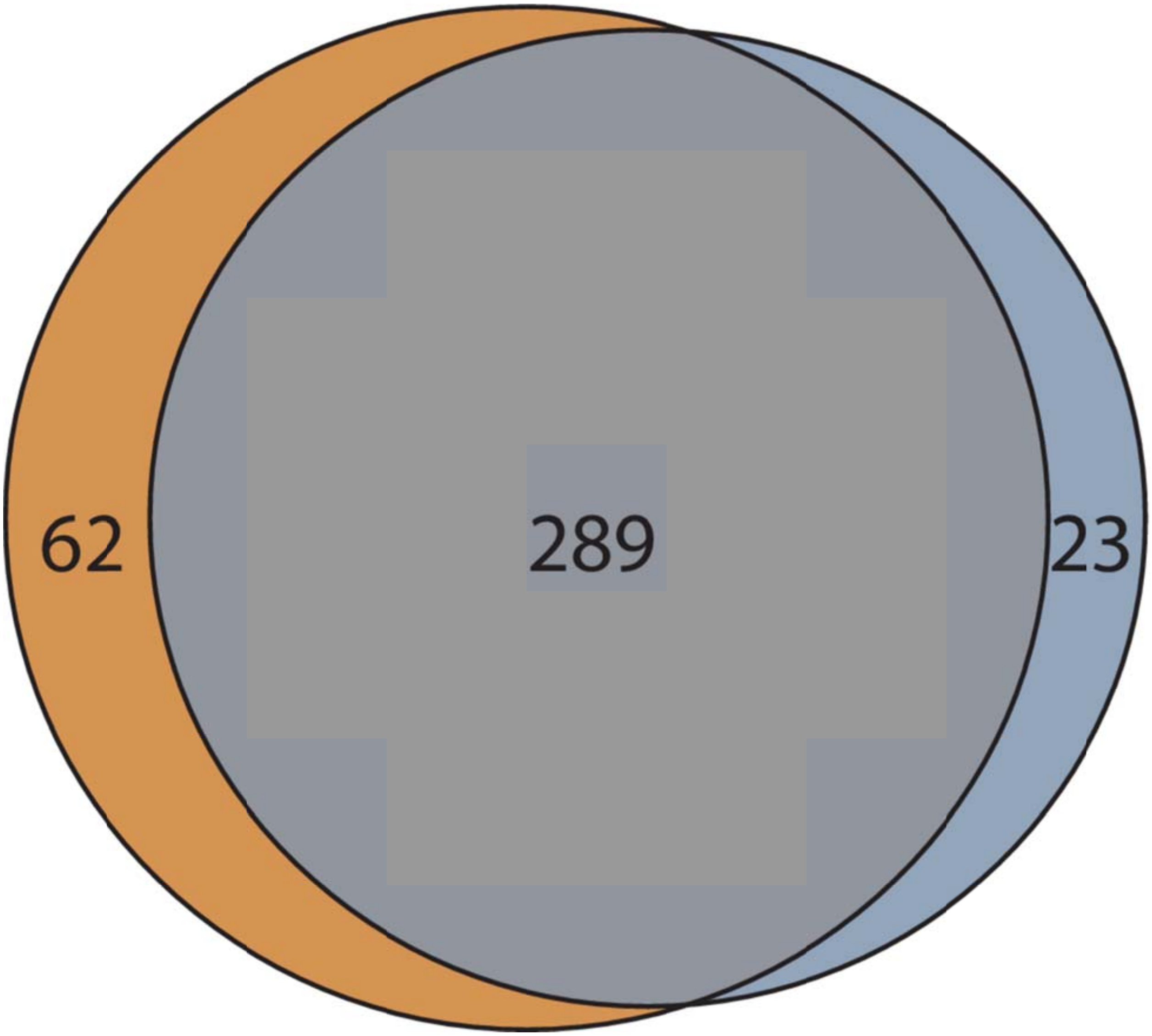

Supplementary Figure 6: Venn diagram comparing the sets of targeted loci that could be amplified in standard, primary RACE (blue) and nested RACE (orange)

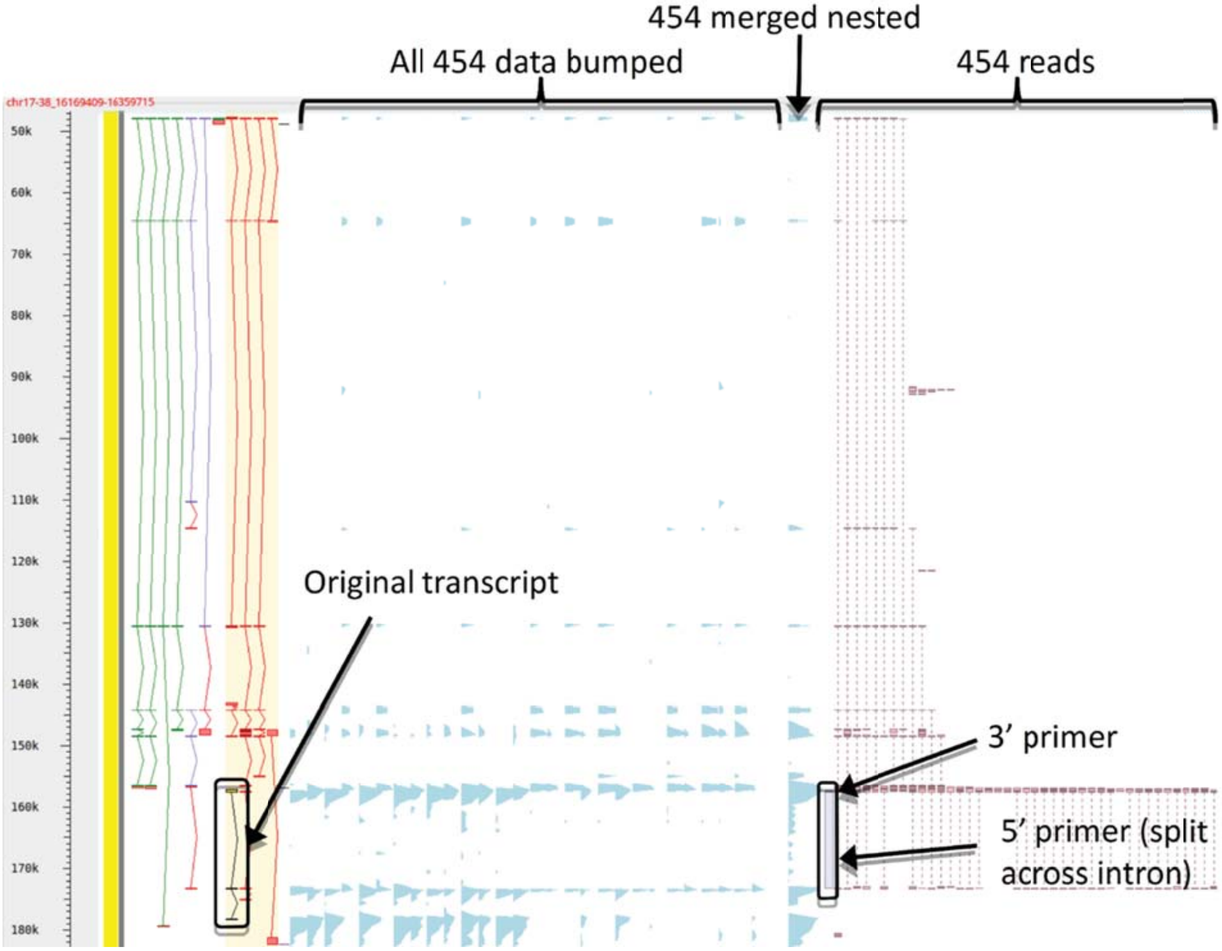

Supplementary Figure 7: Genome browser screenshot of the PIGL locus. The original locus (highlighted, bottom left) was subsumed into the coding locus PIGL. The RNA-seq signal plot (light blue, all 454 data bumped) shows the density of reads mapping to exons. The RACE

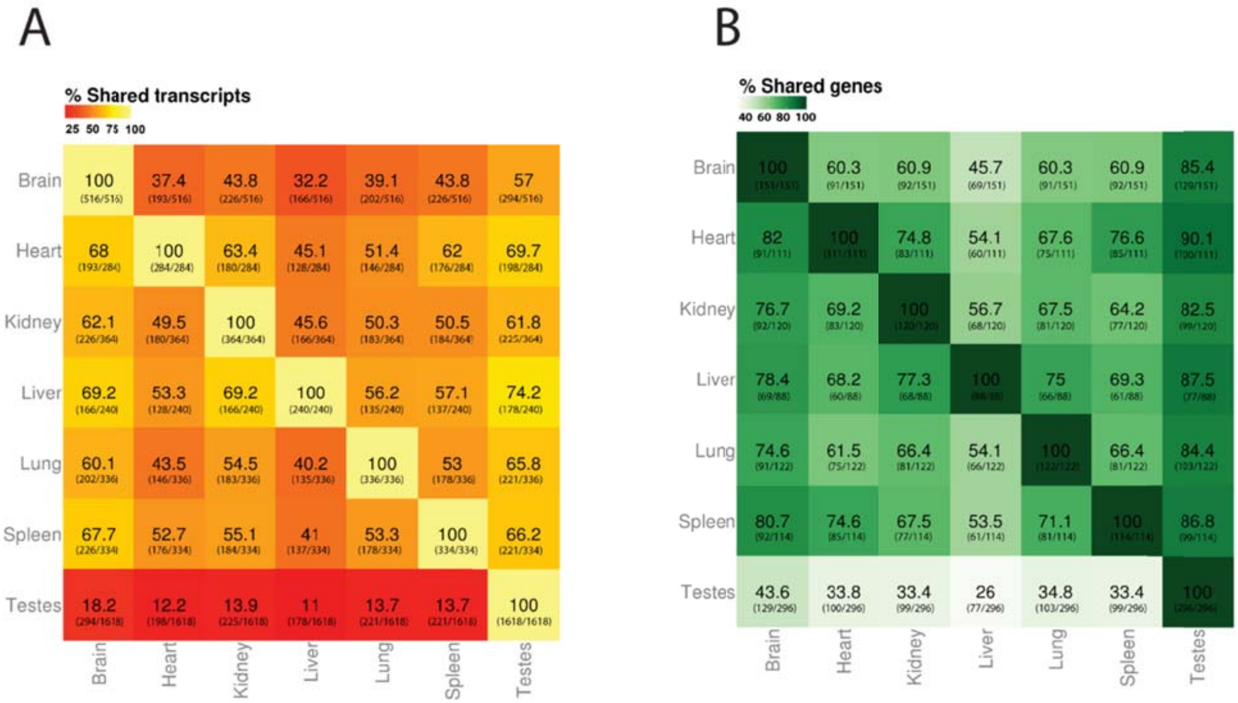

Supplementary Figure 8: Detection of lncRNAs in targeted tissues. Heat map showing the number of detected lncRNAs in each tissue and their proportion shared across other targeted tissues at the (A) transcript level (B) gene level. Transcripts were reconstructed directly from the read alignments, using transcript structure compatibility as merging criterion. A given locus was considered as expressed in a given tissue if at least one of its transcript was successfully reconstructed.

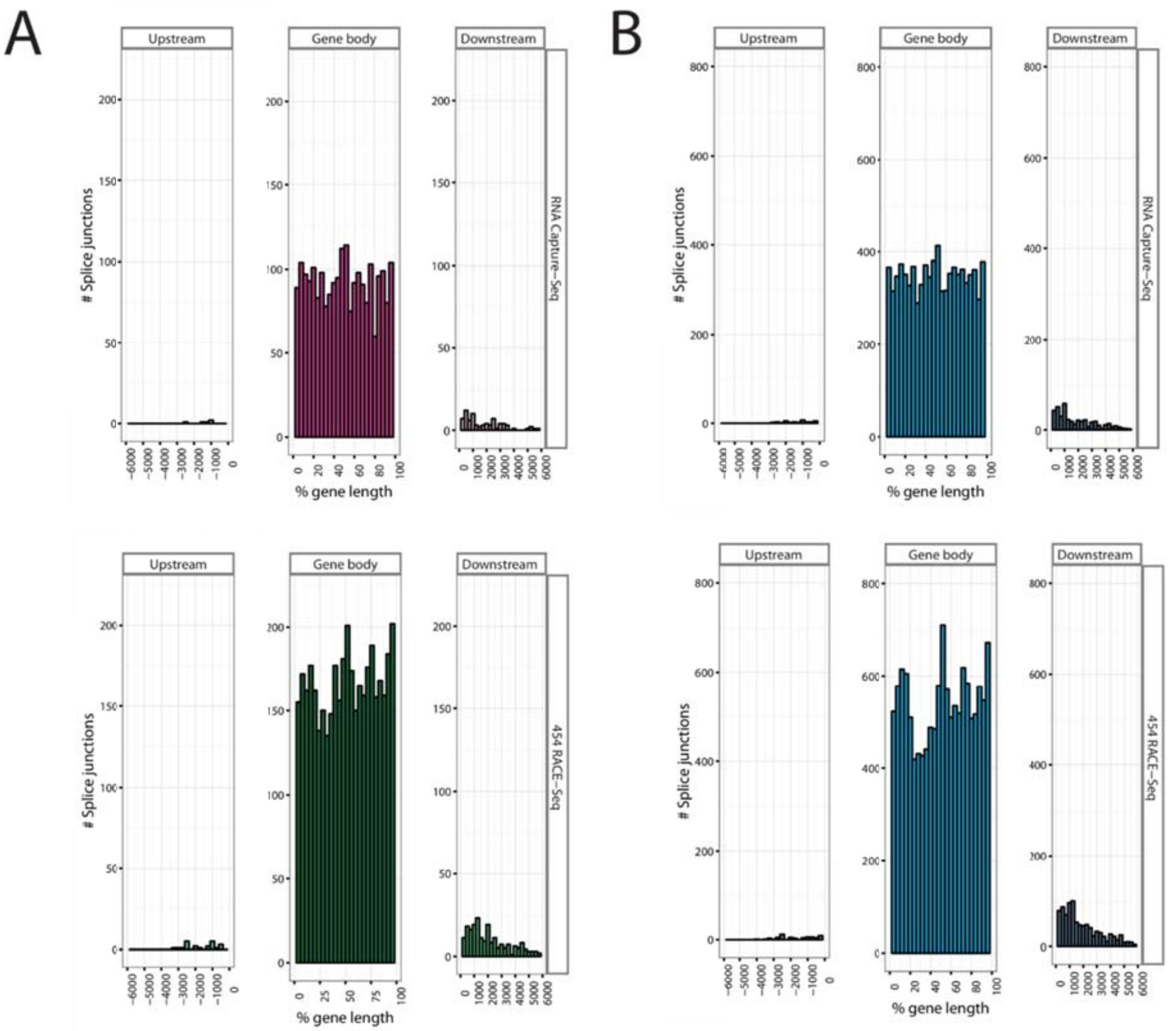

Supplementary Figure 9: Distribution of detected splice junctions by RACE-Seq (bottom panel) and CaptureSeq (top panel) along targeted genes. Bar plots showing the location of splice junctions within targeted loci boundaries (+/- 5kb). (a) All annotated and unannotated splice junctions within targeted loci boundaries (+/- 5kb). ***(A)*** *All annotated and unannotated splice junctions detected in RNA CaptureSeq and RACE-Seq data sets.* ***(B)*** *The top 25% of annotated and unannotated canonical splice junctions ranked by read coverage.*

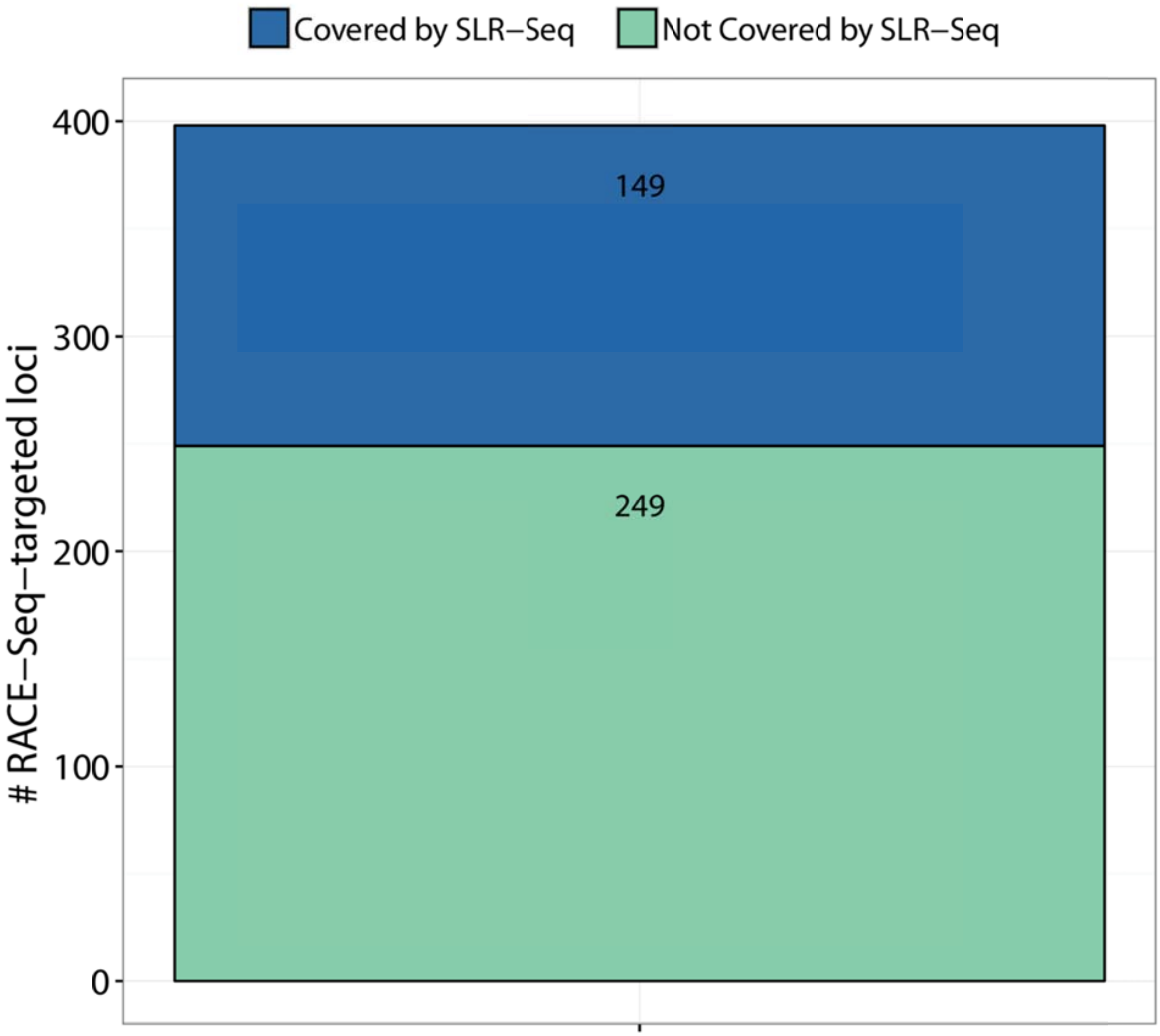

Supplementary Figure 10: Detection of pre- and post-RACE-Seq targets by SLR-Seq. Number of loci covered (blue) and not covered (green) by SLR-Seq reads.

Dataset	Total # TSS (clustered)	# Clustered TSS +/- 50 bp from CAGE tag	% Clustered TSS +/- 50 bp from CAGE tag
**Targets (pre-RACE)**	527	241	46%
**Targets (post-RACE, all)**	873	415	48%
**Targets (post-RACE, novel)**	615	252	41%
**CaptureSeq transcript models (all)**	343	203	59%
**CaptureSeq transcript models (novel)**	70	37	53%

Supplementary Table 1: Table summarizing TSS discovery and CAGE coverage statistics in both RACE-Seq and Clark et al.*'s CaptureSeq.*

Tissue	Pre RACE-Seq (GTEx data)			Post RACE-Seq (standard)				Post RACE-Seq (nested)			
	Total # mapped reads	# reads within targeted transcripts	% reads within targeted transcripts	Total # mapped reads	# reads within targeted transcripts	% reads within targeted transcripts	On-target fold enrichment	Total # mapped reads	# reads within targeted transcripts	% reads within targeted transcripts	On-target fold enrichment
**brain**	37,162,929	330,959	0.9%	746,889	41,019	5.5%	6.2	655,524	328,666	50.1%	56.3
**heart**	35,835,990	564,311	1.6%	857,552	14,475	1.7%	1.1	602,869	171,668	28.5%	18.1
**kidney**	45,384,859	544,064	1.2%	923,780	28,104	3.0%	2.5	730,476	291,291	39.9%	33.3
**liver**	39,432,611	282,230	0.7%	652,592	18,345	2.8%	3.9	751,222	199,511	26.6%	37.1
**lung**	48,531,622	792,644	1.6%	809,139	18,894	2.3%	1.4	834,909	256,859	30.8%	18.8
**spleen**	33,051,498	475,424	1.4%	807,753	16,775	2.1%	1.4	866,228	272,386	31.4%	21.9
**testis**	43,888,261	545,528	1.2%	757,108	76,250	10.1%	8.1	679,735	345,709	50.9%	40.9
**TOTAL**	**283,287,770**	**3,535,160**	**1.2%**	**5,554,813**	**213,862**	**3.9%**	**3.1**	**5,120,963**	**1,866,090**	**36.4%**	**29.2**

Supplementary Table 2: On-target read enrichment statistics. 5' and 3' RACE datasets were merged in each tissue.

# Targets	RACE direction	RACE type	# Targets successfully RACE'd	% Targets successfully RACE'd
398	5'	Standard	248	62%
		Nested	314	79%
		Standard + Nested	341	86%
	3'	Standard	255	64%
		Nested	293	73%
		Standard + Nested	326	82%

Supplementary Table 3: Number and proportion of successfully RACE-amplified targets.

Dataset	Total # unique splice junctions	# Supported by RNA CaptureSeq	% Supported by RNA CaptureSeq
**Targets (pre-RACE)**	1,093	903	83%
**Targets updated (post-RACE)**	3,664	2,211	60%

Supplementary Table 4: Detection of pre- and post-RACE-Seq targets by RNA CaptureSeq. Proportion of annotated splice junctions in pre- and post-RACE-Seq targets supported by RNA CaptureSeq.

Dataset	Total # unique splice junctions	# Supported by 454-RACE-Seq	% Supported by 454-RACE-Seq	# Supported by SLR-Seq	% Supported by SLR-Seq
**Targets (pre-RACE)**	1,093	817	74.8%	226	20.68%
**Targets updated (post-RACE)**	3,664	3,277	89.4%	281	9.11%

Supplementary Table 5: Comparison of splice junction support by 454 RACE-seq and SLR-seq.
